# Effect of Tourniquet Deflation on Intracranial Pressure Measured by Ultrasound of the Optic Nerve Sheath Diameter in Patients Undergoing Orthopedic Surgery Under Spinal Anesthesia: An Observational Study

**DOI:** 10.7759/cureus.46700

**Published:** 2023-10-09

**Authors:** Yellaboina Venkateswarlu, Pratap Rudra Mahanty, Seelora Sahu, Prashant Sharma, Deb Sanjay Nag

**Affiliations:** 1 Department of Anaesthesiology, Tata Main Hospital, Jamshedpur, IND; 2 Department of Anaesthesiology, Manipal Tata Medical College, Jamshedpur, IND

**Keywords:** orthopedic surgeries, spinal anesthesia, tourniquet, intracranial pressure, optic nerve sheath diameter

## Abstract

Background

Orthopedic surgeries of the lower extremities frequently require exsanguination and the use of pneumatic tourniquets. However, the deflation of the tourniquet is accompanied by predominant metabolic changes such as an increase in PaCO_2_. Prior studies have reported the existence of a correlation between tourniquet deflation and an increase in intracranial pressure in patients undergoing surgery under general anesthesia. However, there is a dearth of literature demonstrating such relationships among patients undergoing surgery under subarachnoid block in the Indian setting. The present research was conducted to study the variations in intracranial pressure after the deflation of the tourniquet by measuring the optic nerve sheath diameter (ONSD) using ultrasound among patients undergoing orthopedic surgery of the lower limb under spinal anesthesia at a tertiary care hospital in eastern India.

Methodology

After obtaining clearance from the Institutional Ethics Committee, this prospective observational study was conducted among 45 patients undergoing orthopedic surgeries of the lower limb using a pneumatic tourniquet. Changes in intracranial pressure following tourniquet deflation were recorded by measuring ONSD by ultrasound in these patients. Heart rate (HR), mean arterial pressure (MAP), SpO_2_, EtCO_2_, and ONSD were noted 15 minutes before administration of subarachnoid block (T0), just before tourniquet deflation (T1) and at 5, 10, and 15 minutes after tourniquet deflation (T5, T10, and T15, respectively).

Results

The ONSD varied significantly at each point of observation (p < 0.05). The ONSDs at 5 and 10 minutes after the deflation of the tourniquet were significantly greater than that at T0 (p = 0.002). EtCO_2_ showed a significant increase compared to baseline values at every point of observation intraoperatively whereas MAP showed a significant decrease (p < 0.05). For all parameters (ONSD, HR, systolic blood pressure, diastolic blood pressure, MAP, and EtCO_2_), the most significant change in observation was noted at T10, i.e., 10 minutes after the deflation of the tourniquet.

Conclusions

The significant finding in this study was that the ONSD measurements recorded by ultrasound were increased after the deflation of the tourniquet and that this change can be attributed to an increase in EtCO_2_. However, the results obtained cannot be validated outside the present research owing to the observational nature of the study and limited sample size. Thus, it is difficult to arrive at a definitive conclusion. Further large-scale multicentric studies may be needed to substantiate the findings of this study.

## Introduction

Throughout the world, tourniquets are routinely and safely used during limb surgeries [1]. Due to their benefits in enhancing operating conditions, pneumatic tourniquets are commonly utilized in orthopedic procedures, especially when treating long bone fractures, which make up the bulk of cases [2]. Using a tourniquet during orthopedic procedures creates a bloodless surgical site, enhances vision, protects against surgical complications, and speeds up the procedure, reducing the length of the patient’s hospital stay [3-6].

However, using a tourniquet can lead to several complications, both local and systemic. These concerns include mechanical compression of the underlying structures, ischemia, and reperfusion effects [2]. High cuff pressure and prolonged tourniquet application can lead to local and systemic consequences including nerve damage, vascular injuries, ischemia/reperfusion injury, and raised intracranial pressure (ICP) [3]. Even though they are uncommon, they can have serious impacts, lengthening hospital stay, and may even result in permanent loss of function or limb damage [2].

When the tourniquet is deflated, the increase in carbon dioxide (CO_2_) from the released ischemic metabolic products during the reperfusion period causes increased PaCO_2_, which simultaneously causes cerebral vasodilatation and an increase in cerebral blood flow (CBF), ultimately leading to an increase in ICP [7]. While this transient increase is well tolerated in healthy people, in patients who have a space-occupying lesion with decreased intracranial compliance, it may cause an abrupt rise in ICP [8,9]. Even in individuals with severe brain injury, the dangerous rise in ICP with tourniquet deflation can have serious side effects by producing cerebral volume shifts and possibly irreversible brain damage [3]. Additionally, anesthetic agents can have an impact on ICP during surgery [10].

In the clinical setting, ICP is monitored using both invasive and non-invasive techniques. The gold standard for ICP monitoring is through the intraparenchymal probe or intraventricular catheter, despite being an invasive method [9]. However, the drawbacks of the invasive approach are that it cannot be performed quickly at the bedside, cannulation is difficult and requires expertise, and it is associated with several problems, including infection, trauma, and bleeding [11,12]. The ultrasonographic measurement of optic nerve sheath diameter (ONSD), one of the many non-invasive techniques for measuring ICP, has been accepted as a suitable bedside substitute for invasive techniques [13]. An excellent association has been found between invasive approaches for ICP monitoring and ONSD ultrasonography in earlier investigations [14], which is the reason we selected this method of ICP measurement in our study. Although studies have measured ONSD under general anesthesia, there is a paucity of literature when surgery is done under a subarachnoid block.

## Materials and methods

The study was a hospital-based, prospective, observational study conducted over a period of one year in the Department of Anaesthesia, Tata Main Hospital, Jamshedpur after approval by the Institutional Ethics Committee (IEC), Tata Main Hospital (approval reference number TMH/AC/IEC/NOV/022/2020). A total of 45 patients aged between 18 and 65 years, belonging to the American Society of Anesthesiologists (ASA) Physical Status Classification System grade I-II, and scheduled to undergo elective orthopedic surgery on a lower extremity using a tourniquet under spinal anesthesia were included in this study. Patients aged 65 years or more; those who required bilateral tourniquet usage; those with a history of orbital trauma, optic nerve pathology, glaucoma, asthma, or coronary obstructive pulmonary disease; those with a previous corneal or intraocular surgery; patients with increased ICP, coagulopathy, and local site infection; and those refusing to participate in the study were excluded. Further, patients in whom conversion to general anesthesia was required for any reason perioperatively were also excluded from the study.

All patients underwent standard ASA monitoring including electrocardiogram (ECG), noninvasive blood pressure (NIBP), SpO_2_, and EtCO_2_. This was followed by performing neuraxial block (spinal anesthesia) using a 3 mL dose of 0.5% bupivacaine at the level of L3-L4 or L4-L5, as suitable, and simultaneously co-loading with 1 L of Ringer’s lactate solution. In all patients, a standard pneumatic tourniquet with an 11-cm-wide cuff was placed with the distal edge 15 cm proximal to the proximal pole of the patella, and it was inflated to an inflation pressure of a pneumatic tourniquet pressure which is equal to systolic blood pressure (SBP) + 100 mmHg.

**Figure 1:**
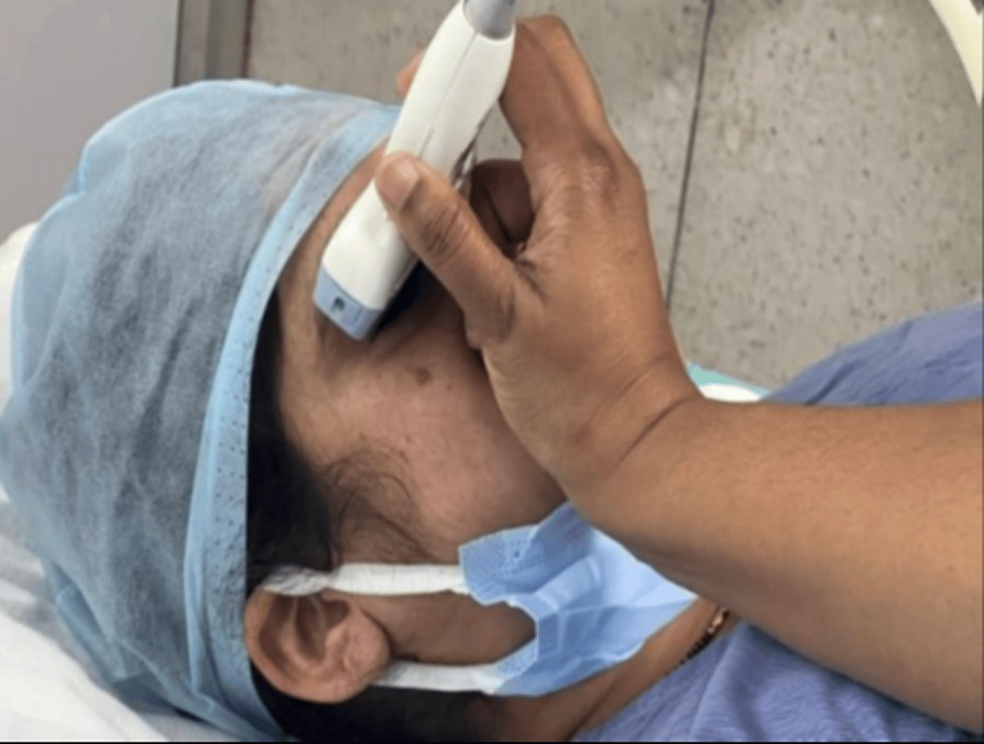
Placement of the ultrasound probe for the measurement of the optic nerve sheath diameter.

**Figure 2:**
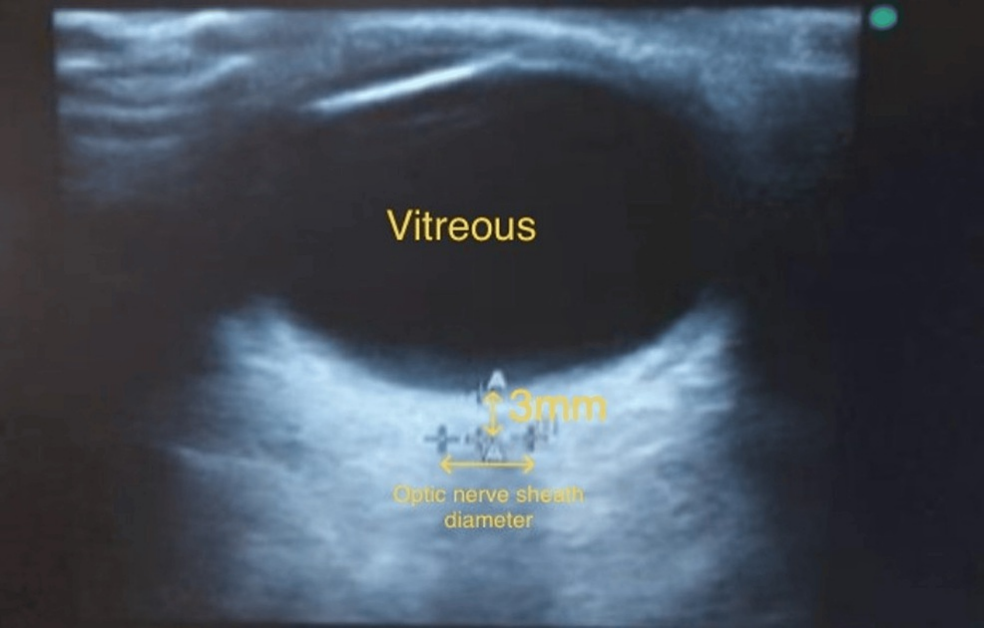
Measurement of the optic nerve sheath diameter by ultrasound.

Sonographic measurement of ONSD (M-Turbo, Sonosite Fujifilm 2016) for all patients was performed by a single anesthesiologist with an experience of at least more than 25 ultrasound measurements. A thin gel layer was applied to the upper eyelid with the patient in the supine position and with the eyes closed (Figure 1). Using a 6-13 MHz linear probe ONSD was measured 3 mm behind the optic globe, with a total of three measurements obtained from each eye on the transverse plane (Figure 2). The final ONSD value accepted was the mean of the three measurements. In many studies, an ICP value of over 20 mmHg has been considered to represent intracranial hypertension. The optimal cut-off value for ONSD is ≥5.0 mm [13]. Heart rate (HR), mean arterial pressure (MAP), SpO_2_, EtCO_2_, and ONSD values were recorded 15 minutes before the administration of subarachnoid block (T0), just before the deflation of the tourniquet (T1) and at 5, 10, and 15 minutes after the deflation of the tourniquet (T5, T10, and T15, respectively). Age, sex, body mass index (BMI), ASA score, and duration of tourniquet use were also noted for each patient. All details were recorded in a predesigned, pretested case record form.

Statistical analysis

The collected data were organized and tabulated in Microsoft Excel 2016 (Microsoft Corp., Redmond, WA, USA) and statistical analysis was done using SPSS version 16.0 (SPSS Inc., Chicago, IL, USA). The data were analyzed by appropriate statistical tools and represented as tables, graphs, diagrams, etc. Continuous variables were expressed as mean ± standard deviation (SD), and categorical variables were expressed as relative frequency and percentage.

Significant changes in ONSD, HR, SBP, DBP, MAP, and EtCO_2_ with different time points were assessed using the Friedman test. Pairwise significant changes in ONSD and EtCO_2_ at different time points (i.e., T1, T5, T10, T15) were assessed using the Wilcoxon signed-rank test. Correlation between ONSD at different time points and EtCO_2_ at different time points was assessed using Spearman’s correlation test. All tests were two-sided. A p-value <0.05 was considered statistically significant.

## Results

The final analysis was done on 45 study participants. The mean age of the study participants was 39.7 ± 11.2 years with a male-to-female ratio of 2.75:1. More than half of the study participants did have any associated comorbidity (57.8%). The proportion of subjects having diabetes only, hypertension only, and both diabetes and hypertension were 4.4%, 31.1%, and 6.7% respectively. The majority of the subjects belonged to the ASA grade I Physical Status classification (73.3%), and the mean body weight was 68.31 ± 7.06 kg. Spinal anesthesia was introduced at the intervertebral level of L3-L4 for 15.6% of the patients and at the level of L4-L5 for the remaining 84.4% of the patients. The mean dose of anesthesia used was 3.02 ±0.19 mL. The mean tourniquet pressure at the time of surgery was 309.33 ± 17.89 mmHg, and the mean duration of tourniquet application was 98 ± 12.0 minutes.

The temporal changes in ONSD, HR, SBP, DBP, MAP, and EtCO_2_ were significantly different at each point of measurement (p < 0.05), as shown in Table 1.

**Table 1:** Temporal changes of important intraoperative parameters (N = 45). *: P-values <0.05 were considered statistically significant. SD = standard deviation; T0 = values at 15 minutes before the induction of anesthesia; T1 = values just before the deflation of the tourniquet; T5 = values at five minutes after the deflation of the tourniquet; T10 = values at 10 minutes after the deflation of the tourniquet; T15 = values at 15 minutes after the deflation of the tourniquet; ONSD = optic nerve sheath diameter (mm); HR = heart rate (beats/minute); SBP = systolic blood pressure (mmHg); DBP = diastolic blood pressure (mmHg); MAP = mean arterial pressure (mmHg); EtCO_2_ = end-tidal carbon dioxide levels (mmHg)

Parameters	T0 (mean ± SD)	T1 (mean ± SD)	T5 (mean ± SD)	T10 (mean ± SD)	T15 (mean ± SD)	P-value*
ONSD	4.3 ± 0.0061	4.4 ± 0.0059	4.6 ± 0.0087	4.6 ± 0.0065	4.4 ± 0.0053	0.003
HR	77.62 ± 5.49	78.58 ± 5.62	78.58 ± 5.62	85.42 ± 4.32	82.09 ± 4.88	<0.001
SBP	132.04 ± 5.91	129.42 ± 5.35	125.47 ± 5.30	122.93 ± 6.16	129.47 ± 4.76	0.007
DBP	81.42 ± 4.34	78.73 ± 4.42	74.73 ± 4.01	73.56 ± 4.92	70.07 ± 3.71	0.041
MAP	98.40 ± 3.65	95.67 ± 3.71	91.69 ± 3.72	90.04 ± 4.42	95.84 ± 3.33	0.036
EtCO_2_	32.71 ± 1.77	34.33 ± 1.62	35.47 ± 2.02	36.58 ± 2.20	34.53 ± 1.78	0.025

Pairwise analysis between the measurement of ONSD and EtCO_2_ at baseline T0 (just before induction) and at other points of observation (T1, T5, T10, T15) showed significant variations in ONSD at each point of observation (p < 0.05). The ONSD at T5 and T10 were significantly greater than that at T0 (p = 0.002), as shown in Table 2.

**Table 2:** Comparison of ONSD and EtCO2 at each point of observation with baseline measurement through pairwise analysis (N = 45). *: P-values <0.05 were considered statistically significant. T0 = values at 15 minutes before the induction of anesthesia; T1 = values just before the deflation of the tourniquet; T5 = values at five minutes after the deflation of the tourniquet; T10 = values at 10 minutes after the deflation of the tourniquet; T15 = values at 15 minutes after the deflation of the tourniquet; ONSD = optic nerve sheath diameter (mm); EtCO_2_ = end-tidal carbon dioxide levels (mmHg)

Parameters	T0	T1	T0	T5	T0	T10	T0	T15
ONSD	4.3 ± 0.0061	4.4 ± 0.0059	4.3 ± 0.0061	4.6 ± 0.0087	4.3 ± 0.0061	4.6 ± 0.0065	4.3 ± 0.0061	4.4 ± 0.0053
P-value	0.032*		0.002*		0.002*		0.032*	
EtCO_2_	32.71 ± 1.77	34.33 ± 1.62	32.71 ± 1.77	35.47 ± 2.02	32.71 ± 1.77	36.58 ± 2.20	32.71 ± 1.77	34.53 ± 1.78
P-value	0.044*		0.025*		0.001*		0.036*	

Correlation between differences in ONSD and EtCO_2_ analyzed at different points of observation intraoperatively showed that although there was a weak negative correlation observed between ONSD and EtCO_2_ at T0, T1, T5, and T15, none of the associations were statistically significant (p > 0.05). The best correlation between ONSD and EtCO_2_ was noted at T10 (r = -0.15, p = 0.033).

## Discussion

The use of pneumatic tourniquets in orthopedic surgery provides the benefits of reduced operating time and lesser blood loss during the procedure. The pH, arterial oxygen partial pressure (PaO_2_), arterial carbon dioxide partial pressure (PaCO_2_), potassium ion, and lactate levels fluctuate because of tourniquet inflation and deflation [15]. Tourniquet deflation results in the release of carbon dioxide (CO_2_) into the systemic circulation that has collected during the ischemia phase. A rise in ICP follows the resulting elevation in PaCO_2_. Although most healthy patients can compensate for and tolerate this increase in CBF, it may result in an increase in ICP in patients with impaired intracranial compliance [15]. Cerebral ischemia is one of the harmful effects of elevated ICP. Therefore, patients undergoing lower limb surgery who have or are at high risk of cerebrovascular illness require careful monitoring and meticulous treatment of the ICP utilizing a pneumatic tourniquet [15].

The intraventricular catheter and the intraparenchymal catheter, which are regarded as the gold standard approaches based on their accuracy, are two techniques that can be used to monitor the ICP in real time [16,17]. These intrusive procedures, however, have the highest risk of consequences and are not always practical [17]. To indirectly estimate ICP, noninvasive techniques such as measuring the ONSD are now preferred [18,19]. In most instances, it is simple to visualize the ONSD. Additionally, an increase in ONSD is a precursor to an increase in ICP, and a rise in ICP causes cerebrospinal fluid to move from the cerebral subarachnoid space into the optic subarachnoid space [20]. Therefore, the ONSD is helpful for keeping an eye on the ICP during surgery [15].

To our knowledge, no research has been done in the Indian setting on the use of ultrasonography to detect an increase in ICP using ONSD after tourniquet deflation during lower extremity procedures. The only similar studies were an observational study conducted in Turkey by Besir et al. published in 2018 and a clinical trial conducted by Kim et al. in South Korea published in 2022.

The present study was conducted to investigate the variations in ICP following tourniquet deflation by measuring ONSD by ultrasound. HR, MAP, ETCO_2_, and ONSD values were recorded 15 minutes before the administration of subarachnoid block (T0), immediately before tourniquet deflation (T1), and at 5, 10, and 15 minutes after tourniquet deflation (T5, T10, and T15, respectively). The temporal changes in ONSD, HR, SBP, DBP, MAP, and EtCO_2_ were significantly different at each point of measurement (p < 0.05). The ONSD showed significant differences at each point (p < 0.05). The ONSDs at T5 and T10 were significantly greater than that at T0 (p = 0.002). This study finding is consistent with that reported by Kim et al. [15] where significant differences in ONSD were noted at each observation point. In our study, the highest difference in ONSD value was noted at T10 (10 minutes after the deflation of the tourniquet), which is contrary to that reported by Kim et al. [15] as well as Besir et al. [13] where the maximum changes in ONSD were observed at five minutes following the deflation of the tourniquet.

Results from our study showed a significant decrease in MAP whereas EtCO_2_ significantly increased compared to baseline values at every point of observation intraoperatively (p < 0.05). For all parameters (ONSD, HR, SBP, DBP, MAP, and EtCO_2_), the highest change in observation was noted at T10, i.e., 10 minutes after the deflation of the tourniquet. The best correlation between ONSD and EtCO_2_ was noted at T10 with a correlation coefficient of r = 0.15, p = 0.033. This finding is similar to that reported by the aforementioned studies, where the change in ONSD was significantly correlated with the change in the EtCO_2_ after tourniquet deflation [13,15]. However, the correlation coefficient obtained in the present study is much weaker than that reported by the other studies.

In many investigations, intracranial hypertension was defined as an ICP value of more than 20 mmHg [13]. The ONSD cut-off setting that works best is 5.0 mm. However, there are several ONSD cut-off values in the literature that range from 5.2 to 5.9 mm and were determined by invasive and noninvasive techniques. The sensitivity and specificity of this parameter ranged from 74% to 95% and 100%, respectively [21-24]. In the present study, the mean ONSD value observed 5 and 10 minutes after deflation was 4.6 mm. These figures are lower than 5.1 mm at five minutes post-deflation reported by Besir et al. [13]. Lower ONSD values in our case compared to Besir et al. may be attributed to the fact that all our cases were performed under spinal anesthesia, thus negating the vasodilatory and ICP-increasing effect of general anesthesia. Our study had the advantage of using neuraxial block compared to inhaled anesthetics used by Besir et al. [13]. Inhaled anesthetics might have an effect on CBF and ICP due to vasodilatation [25]. Thus, observations in our study have not been skewed by not using this potential confounder.

This study did have some limitations. First, the measurement of ICP by invasive methods, such as the placement of an intraventricular catheter, is considered the gold standard. This was not done in this study due to the requirement of invasive surgical techniques and ethical considerations. Second, this research was performed as an observational study only, and further prospective randomized trials in this domain may be required. Lastly, because the study was conducted in a single hospital, it has the disadvantages of a limited sample size and centripetal bias. Further studies in different settings in other parts of the country and on a larger population are necessary to validate our study findings.

## Conclusions

The present study is the only study, to our best knowledge, conducted in the Indian setting to evaluate changes in ICP following tourniquet deflation after orthopedic surgeries of the lower limb under subarachnoid block. The significant finding in this study was that the ONSD measurements obtained by ultrasound were increased following tourniquet deflation and that this change was linked to an increase in EtCO_2_. However, these findings cannot be validated outside the present research owing to the observational nature of the study and limited sample size. Thus, it is difficult to arrive at a definitive conclusion. Further large-scale, multicentric studies may be needed to substantiate the findings of this study.
